# Opium Poisoning

**Published:** 1876-02

**Authors:** J. M. Johnson

**Affiliations:** Atlanta, Ga.


### OPIUM POISONING.

By J. M. JOHNSON, M.D., of Atlanta, Ga.

After the reading of Dr. Todd's essay, at the last meeting of the Academy of Medicine, on the antidotal properties of veratrum veride in opium poisoning, the discussion ended abruptly and unexpectedly; and I did not then call it up, as I might have done, for the reason that I have a case to report that I thought ought to be heard. But I am unwilling that Dr. Todd's paper should go to the profession without the dissent of at least one earnest objector to the doctrine of antidotal relief from opium poisoning.

There is not in the Materia Medica a remedy so complex in its constitution. No less than ten different alkaloids had been discovered when I last examined the subject, every one differing in its reaction and test; and the investigation of the subject only begins with this century. Over twenty different elemental bodies have been found. It was not until 1817 that Searturner discovered the alkaloid which has subsequently been called narcotine. Three other of the alkaloids contain narcotine. They are recognized, 1st, by the peculiar stimulating effects upon the brain; 2d, by drowsiness; and 3d, by stupor and coma.

All the other alkaloids and elemental substances have their peculiar therapeutic effects, but none of them disturb the functions of the brain or cause constipation except those named above. Codia may be named as a type of all the others. While it causes sleep, there is no sudden heavy effect produced upon the brain; nor does it produce constipation, but acts like bromide of potassium, camphor, valerian, sulphuric ether, etc, by soothing the great organ of active life, and giving sweet sleep without dreams or horrible phantasmagoria.

In crude opium all these qualities are combined; but it cannot be denied that in many instances the best effects are obtained from it. It is agreed that opium finds its way into the blood from the stomach—that it comes into contact with the nerve centres and trunks alike; that it first stimulates, then produces drowsiness, then sleep, deep stupor, and in case of poisoning, coma; that the heart beats until the brain stimulus is gone, and then complete asphyxia ensues. The brain dies first, but if the breathing is kept up by artificial means, and the heart continues to send arterial blood to the brain, resuscitation follows, and the brain is alive again.

No traces of inflammation are found on examination of the bodies of those who die. The universal opinion is that death is caused by suspended respiration, from loss of the voluntary functions, and paralysis of the unstriated fibres. Its operation is by immediate contact with the brain, cord, and nerve trunks. The brain has to struggle through its contact with the poison, and hence electricity, artificial respiration, scourging with switches, walking, or the employment of constant manipulations of some kind, are necessary to keep up the circulation until the battle can be fought out by the brain. Coffee has no antidotal property, except its tendency to cause wakefulness, and by its heat and peculiar stimulant properties to restore the functions of the stomach.

By the common testimony of all observers, opium, in remedial portions, produces, 1st, sleep; 2d, relieves pain; 3d, stimulates, diffuses and equalizes the circulating mass; 4th, it is antispasmodic, controlling muscular action and soothing the brain and nerves; 5th, it allays general and local irritation, and, under proper circumstances, inflammation also; 6th, it suppresses morbid discharges; 7th, combined with nauseants it is our best diaphoretic. This great diversity of power is due, undoubtedly, to the large number of alkaloid and elementary substances entering into and forming the constitution of the drug, but, by a law of its nature, acting harmoniously as a whole.

These alkaloids and elementary principles, three-fourths of which we know nothing about, but all of which are doubtless factors in its poisonons as well as in its curative effects, point unerringly to the fact that the antidotal theory is a myth. Men frequently recover after taking large draughts of laudanum and great quantities of opium, and the last thing given is generally regarded as the antidote.

It is a fact very well known, that nearly all the opium on sale throughout the world is more or less adulterated; many times half the mass is made up of cow's dung and other impurities. The opium and tinct. of opium, under such circumstances, would be only half strength. Generally, persons who attempt suicide by taking this drug, have been accustomed to its use, and can tolerate very large quantities; but the habit having been concealed, as is always the case, and no one being aware of it, all are easily missled. The doctors are called in, the man is vomited, if it is possible to do it, without regard to the length of time that has elapsed since the drug was swallowed; belladonna and veratrum veride are injected repeatedly under the skin, cold water is abundantly applied, the battery is brought up and electricity does its share. After frequent repetitions of all this, and more, the man, by a miracle, lives.

On the other hand, if the man (such as I have described) had been vomited with a drachm of alum dissolved in half a pint of hot water, and administered every two minutes, in five minutes everything in his stomach will be thrown out, and you can then commence the keeping-awake treatment, such as giving coffee as hot as can be taken, not only to keep him awake, but also to restore the functions of the stomach, and by its heat and other stimulating qualities prevent congestion of the brain and lungs, by restoring and maintaining capillary action, and to the same end, walking, whipping with fine switches, cold douches to the head, etc. If the breathing is so disturbed as to require Hall's method, then put him on his side and face, in the open air, and keep up respiration, and, as a consequence, the heart's action, so as to furnish arterial blood to the brain to prevent stagnation and death. All the cases that are curable can be cured in this way. But do not use belladonna or veratrum; we do not understand either of these remedies well enough to employ them so rashly; and besides, the tincture of veratrum is powerfully sedative, and will do harm if it does anythiag. Belladonna dilates the pupils, but the fact only is known, while the mode is unknown. It excites skin action, and so does opium.

But it is unnecessary to discuss this. Medical philosophy has taken a stand of late, to accept nothing without good reason therefor. Remember and take courage from it, that half the opium taken may be cow's dung, which some wicked Egyptian, Greek or Hindoo has put into his little batch to increase his profits, but which Providence sanctifies to the advantage of the poor would-be suicide.

