# Errata

**Published:** 1876-02

**Authors:** 


ERRATA.—The following errata occur in the article of Dr. Jno. M. Johnson, on "Opium Poisoning: First paragraph, fifth line, read had for have; third paragraph, fifth line, sodden for sudden; sixth paragraph, second line, transpose the words "sleep" and "stimulates."

